# Down regulation of miR-124 in both Werner syndrome DNA helicase mutant mice and mutant *Caenorhabditis elegans wrn-1* reveals the importance of this microRNA in accelerated aging

**DOI:** 10.18632/aging.100489

**Published:** 2012-09-29

**Authors:** Alexandra Dallaire, Chantal Garand, Eric R. Paquet, Sarah J. Mitchell, Rafael de Cabo, Martin J. Simard, Michel Lebel

**Affiliations:** ^1^ Centre de Recherche en Cancérologie de l'Université Laval, Hôpital Hôtel-Dieu de Québec, Québec City, Québec, G1R 2J6, Canada; ^2^ Laboratory of Experimental Gerontology, National Institute on Aging, National Institutes of Health, Baltimore, MD 21224-6825, USA; ^3^ Sydney Medical School, University of Sydney, Sydney NSW, Australia

**Keywords:** Werner syndrome, aging, microRNA, liver, mouse, nematode

## Abstract

Small non-coding microRNAs are believed to be involved in the mechanism of aging but nothing is known on the impact of microRNAs in the progeroid disorder Werner syndrome (WS). WS is a premature aging disorder caused by mutations in a RecQ-like DNA helicase. Mice lacking the helicase domain of the WRN ortholog exhibit many phenotypic features of WS, including a pro-oxidant status and a shorter mean life span. *Caenorhabditis elegans (C. elegans)* with a nonfunctional wrn-1 DNA helicase also exhibit a shorter life span. Thus, both models are relevant to study the expression of microRNAs involved in WS. In this study, we show that miR-124 expression is lost in the liver of Wrn helicase mutant mice. Interestingly, the expression of this conserved miR-124 in whole *wrn-1* mutant worms is also significantly reduced. The loss of *mir-124* in *C. elegans* increases reactive oxygen species formation and accumulation of the aging marker lipofuscin, reduces whole body ATP levels and results in a reduction in life span. Finally, supplementation of vitamin C normalizes the median life span of *wrn-1* and *mir-124* mutant worms. These results suggest that biological pathways involving WRN and miR-124 are conserved in the aging process across different species.

## INTRODUCTION

Aging is a progressive deterioration of physiological functions impairing the ability of an organism to cope with endogenous or exogenous stresses and maintain homeostasis. This in turn leads to increased susceptibility to diseases and death. The study of human progeroid syndromes have greatly advanced the understanding of the aging process [1]. One fascinating human aging disorder is Werner syndrome (WS). WS is a human autosomal recessive disorder characterized by genomic instability and the premature onset of a number of age-related diseases [2-5]. The defective enzyme responsible for WS possesses a 3′–5′ exonuclease activity in addition to a 3′–5′ helicase activity [6-8] and is involved in DNA repair, replication, transcription, and telomere maintenance [9-13]. We previously generated a mouse model with a deletion in the helicase domain of the murine *WRN* homologue (hereafter referred as *Wrn^Dhel/Dhe^l*) [14] that recapitulates most of the WS phenotypes, including an abnormal hyaluronic acid excretion, higher reactive oxygen species (ROS) levels, dyslipidemia, increased genomic instability, and cancer incidence. Overall, such mutant mice have a 10-15% decreased of their mean life span [15, 16].

The WRN protein is a member of the RecQ family of DNA helicases [4]. It is highly conserved across species including in invertebrates such as the small worm *Caenorhabditis elegans (C. elegans)*. Interestingly, the exonuclease and the DNA helicase domains homologous to the human WRN protein are encoded by two different genes in *C. elegans* [17]. The *C. elegans wrn-1* gene codes for the ATP-dependent 3′–5′ DNA helicase capable of unwinding a variety of DNA structures [18]. Notably, it has been shown that the RNAi knockdown of the *C. elegans wrn-1* gene shortens the life span, increases sensitivity to DNA damage, and accelerates aging phenotypes [17].

Recent discoveries in the fields of development, cancer, and aging have indicated that small non-coding RNAs play a major role in alterations associated with these biological processes. An important class of non-coding RNAs that has been studied in the context of *C. elegans* aging are the microRNAs (miRNAs) [19-23]. The miRNAs are short RNAs (~22nt) that regulate post-transcriptional gene expression via base pairing to partially complementary sites mainly found in the 3’ UTRs of messenger RNAs (mRNAs). miRNAs down regulate protein expression by inhibiting mRNA translation and/or mRNA stability [20]. Individual miRNAs can modulate multiple mRNA targets, and individual mRNAs can be regulated by multiple, distinct miRNAs [20]. Very few studies using rodent tissues have been performed to elucidate the role of miRNAs in aging [24-27], often with contradictory results [28, 29].

In this study, we report the differential expression of several miRNAs in the livers of young (three months old) *Wrn^Δ^^hel/^^Δ^^hel^* mice compared to age-matched wild type animals. Among them, one conserved miRNA in animals (miR-124) was down regulated in both the liver of *Wrn^Dhel/Dhel^* mice and in whole wrn-1 *C. elegans* mutants. Deletion of mir-124 in C. elegans resulted in a decrease in life span, an increase in reactive oxygen species (ROS) production, a decrease in ATP levels, and an increase in the aging marker lipofuscin. All these phenotypes could be reversed in mir-124 mutation strains after vitamin C treatment. These results implicate a role for the conserved miR-124 in aging in *C. elegans*.

## RESULTS

### The liver of *Wrn^Δ^^hel/^^Δ^^hel^* mice show differential expression of miR-375 and miR-124

We have previously shown that in *Wrn^Δ^^hel/^^Δ^^hel^* mice, the liver is the first tissue to show morphological changes compared to age-matched wild type animals [16, 30]. Interestingly, the liver undergoes substantial modifications in structure and function in old age including include alterations in liver mass, blood flow, and sinusoidal cell morphology [31]. These changes are associated with significant impairment of many hepatic metabolic and detoxification activities, with implications for systemic aging and age-related disease. We therefore focused our study on the hepatic tissue as the liver plays a pivotal role in whole body homeostasis through the maintenance of nutrient, drug, hormone, and metabolic processes. Total RNA from the liver of two *Wrn^Dhel/Dhel^* and two wild type mice at three months of age was extracted to analyze the expression of 755 different miRNAs using the TaqMan-based Array. Although no gross hepatic morphological difference could be observed between *Wrn^Dhel/Dhel^* mice and wild type mice at three months of age, the liver of *Wrn^Dhel/Dhel^* mice exhibited changes in the expression of a number of miRNAs compared to wild type mice. Supplementary Tables S1 and S2 provide the raw data on all miRNAs. Table 1 summarizes the list of differentially expressed miRNAs in the liver of *Wrn^Dhel/Dhel^* mice compared to wild type mice.

**Table 1:** List of miRNAs differentially expressed in the liver of three months old *Wrn*^Δ*hel/*Δ*hel*^ mutant compared to wild type mice with an adjusted *P*-value < 0.1

miRNA	expression*	*P*-value	Adjusted *P*-value
let-7i	down	0.000058	0.0071
miR-350	down	0.000250	0.0153
miR-2183	up	0.000386	0.0157
miR-375	up	0.000249	0.0362
miR-15a	down	0.002092	0.0638
miR-124	down	0.000883	0.0641
miR-509-3p	down	0.001641	0.0641

* Expression of miRNAs in the liver of *Wrn*^Δ*hel/*Δ*hel*^ mutant compared to wild type mice.

**Figure 1:**
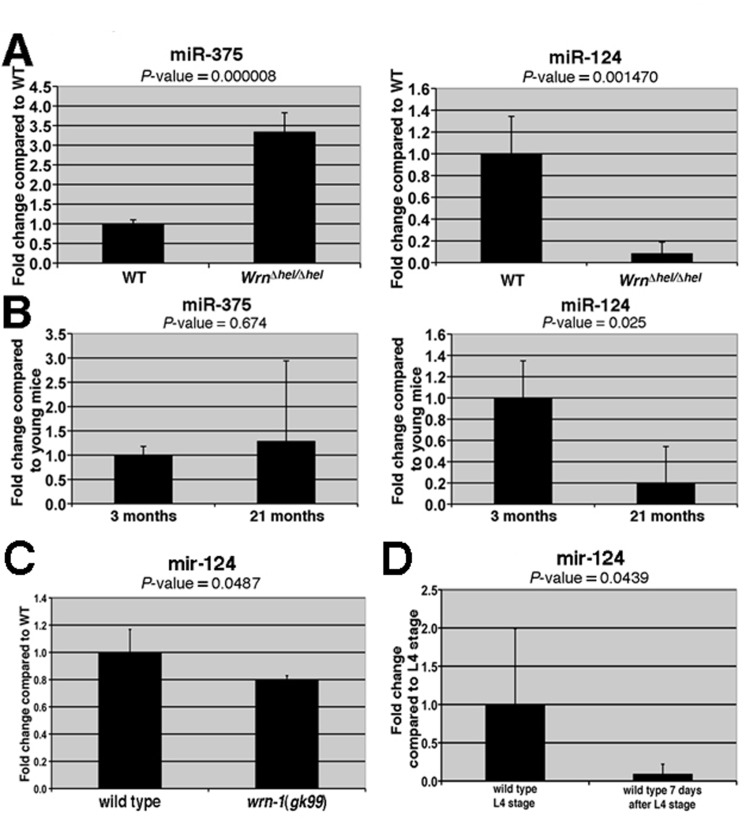
Expression levels of miRNAs in the liver of *Wrn^Dhel/Dhel^* mice compared to wild type mice and in the whole body of wild type and *wrn-1(gk99)* worms. (**A**) Total RNA from four mice (at three months of age) of the indicated genotype was used for the quantitative RT-PCR analyses. The levels of the indicated miRNAs in the *Wrn^Dhel/Dhel^* mice are relative to the wild type (WT) animals. (**B**) Expression levels of miR-375 and miR-124 in the liver of four young (three months old) and four old (21 months old) wild type animals. The levels of the indicated miRNAs in the old wild type mice are relative to the young wild type animals. (**C**) Expression level of *mir-124* in wild type and *wrn-1(gk99)* strains. Twenty-five 7-day old adult worms (post-larval L4 stage) of each strain were sorted and collected for total RNA extraction. The quantification of *mir-124* were measured by quantitative RT-PCR (TaqMan assay) and compared with the levels found in wild type animals. (**D**) *mir-124* expression levels in young and older adults wild type worms. All data were normalized by the quantification of the small nucleolar RNA (*sn2841*). The error bars represent the 95% confidence interval of three independent experiments. The *P*-values (unpaired Student's t-test) are indicated above each graph.

We next validated the differential expression of the seven miRNAs listed in Table 1 using the liver tissues of four different *Wrn^Dhel/Dhel^*mutant and four wild type mice (three months of age) Of the seven miRNAs tested, only miR-375 and miR-124 showed significant differential expressions in *Wrn^Dhel/Dhel^* mutant compared to wild type animals (Figure 1A and Supplementary Figure S1). miR-375 was up regulated more than three-fold and miR-124 was down regulated by ten-fold in the liver of *Wrn^Dhel/Dhel^* mutant mice compared to the liver of wild type animals (Figure 1A).

To determine whether miR-375 and miR-124 were also differentially expressed during aging, quantitative RT-PCR was performed on the liver tissues of four young (three months) and four old (21 months) wild type mice. miR-124 was significantly decreased (by five-fold) in the livers of old wild type mice compared to young wild type mice (Figure 1B). In contrast, there was a non-significant increase in miR-375 level in the liver of old wild type animals. These results indicate that the expression of miR-124 correlates inversely with age in the liver of mice (Figure 1B).

### The impact of the Wrn helicase on miR-124 expression is conserved in *C. elegans*

**Figure 2:**
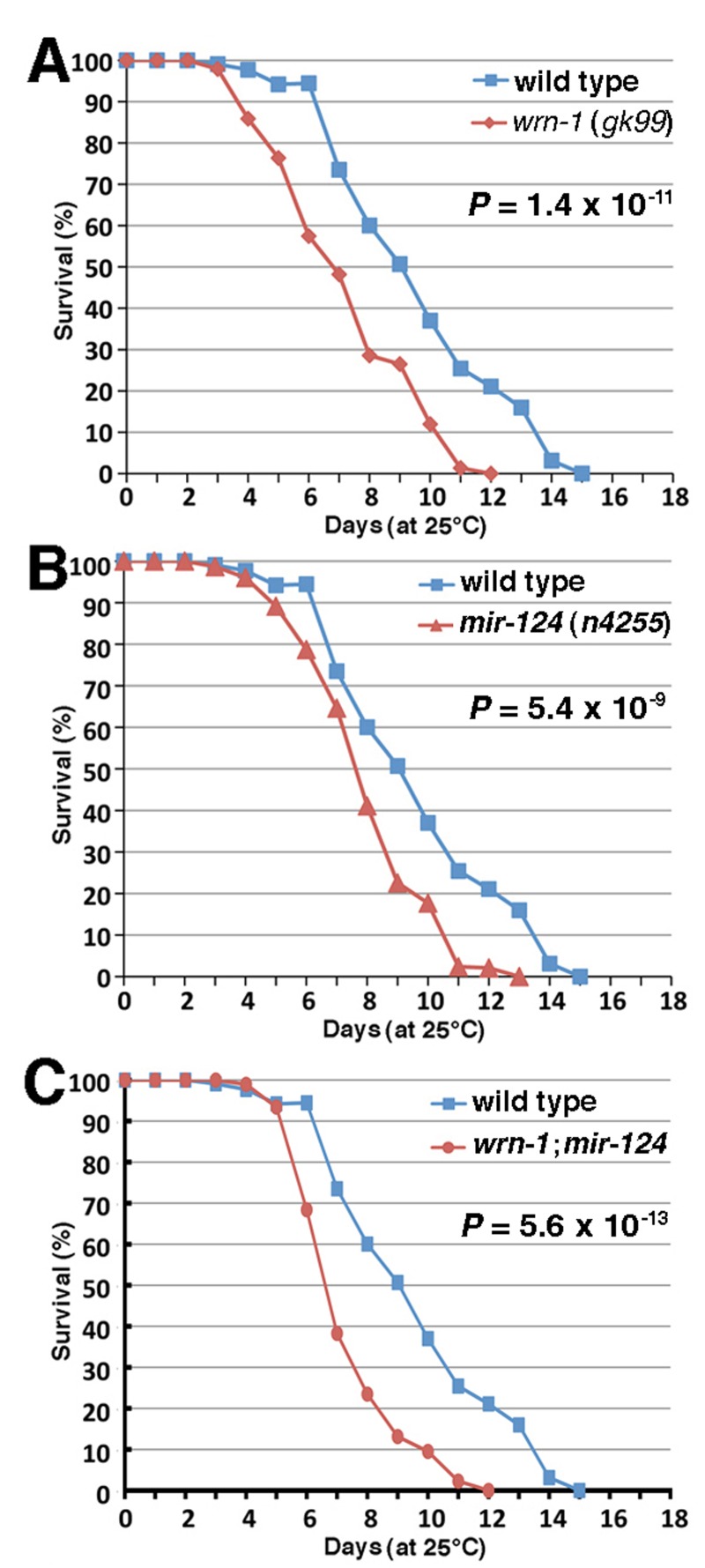
Life span of wild type and mutant *C. elegans animals*. (**A**) Survival curves of wild type (N2) and *wrn-1(gk99)* animals grown at 25°C. (**B**) Survival curves of wild type (N2) and *mir-124 (n4255)* strains grown at 25°C. (**C**) Survival curves of wild type (N2) and *wrn-1;mir-124* double mutant animals grown at 25°C. All experiments were performed with five different pools of 20 to 30 animals of each genotype. The indicated *P*-values were obtained using the log-rank test method.

We next determine whether the observed alteration of the miRNAs in mice could be a global phenomenon during aging by studying these miRNAs in the nematode *C. elegans*. A search in the miRNA database miRBase (www.mirbase.org) revealed that miR-124 is conserved in the short-lived *C. elegans* but not the miR-375. We first determined if the modulation of miR-124 is also conserved in *C. elegans* animals carrying a loss-of-function deletion of the *wrn-1 gene (wrn-1(gk99) allele)* that encodes the human WRN helicase ortholog [32]. It has been reported that a depletion of the *C. elegans wrn-1* gene product by RNAi reduces the life span of this animal [17]. Consistent with these findings, we found that the *wrn-1(gk99)* mutant animals had a reduced life span when compared to the wild type (N2) animals (Figure 2A). The median life span of the *wrn-1(gk99)* animals was 6.8 days compared to 9.0 days for the wild type strain (32% decrease; log-rank test: P-value = 1.4 × 10^−11^). Interestingly, we observed that the expression of the conserved miR-124 is significantly reduced by 20% in the *wrn-1(gk99)* animals (unpaired Student's *t-test: P* = 0.048) compared to the wild type strain (Figure 1C). Furthermore, we found that *mir-124* expression is also reduced in older wild type worms (seven days after L4 stage) compared to young worms (at the L4-larvae developmental stage) (Figure 1D). These results indicate that miR-124 expression is decreased in both *Mus musculus* and *C. elegans* during aging and in animals with a mutation in the *WRN* helicase ortholog.

### The loss of mir-124 causes a reduction of life span in*C. elegans*

To assess the impact of the loss of miR-124 on aging, we measured the life span of worms carrying a deletion of the*mir-124* gene (*mir-124(n4255)*) [33]. As shown in Figure 2B, the median life span of *mir-124(n4255*) worms was significantly decreased by 15% (7.7 days *versus* 9.0 days) compared to the wild type animals (log-rank test: *P* = 5.4 × 10^−9^). Notably, animals carrying both deletion of *mir-124* and *wrn-1* genes (*wrn-1;mir-124* animals) displayed a more severe decrease in their life span (48% decrease compared to wild type; log-rank test: *P* = 5.6 × 10^−13^) than the single loss of either gene (Figure 2C). These results indicate that both genes are important in the life span of *C. elegans*.

### The loss of *wrn-1* and *mir-124* leads to an increase in reactive oxygen species (ROS) generation and a reduction in ATP levels

**Figure 3:**
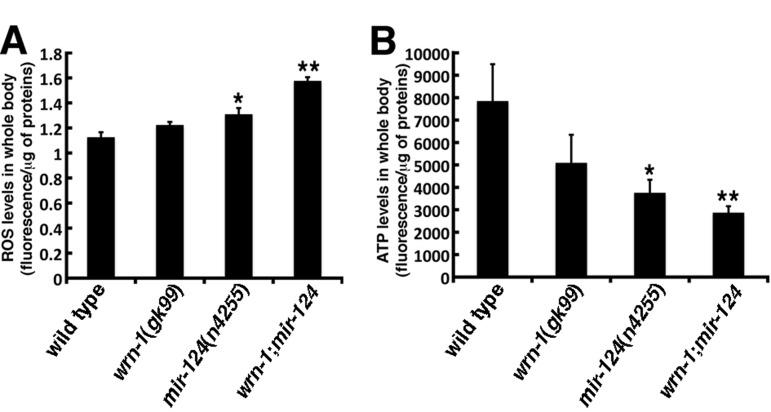
Reactive oxygen species (ROS) and ATP levels in wild type and mutant *C. elegans* strains. (**A**) ROS levels in whole body of 7-day adult old worms (post-larval L4 stage) detected with dichlorofluorescein. Data are expressed as units of fluorescence per micrograms of proteins. (Unpaired Student's t-test; *P = 0.0442 vs. wild type; **P = 0.0008 vs. wild type). (**B**) ATP levels in whole body of 7-day old adult worms (post-larval L4 stage). (Unpaired Student's t-test; *P = 0.0410 vs. wild type; **P = 0.0137 vs. wild type). Data are expressed as units of fluorescence per micrograms of proteins. Twenty-five worms of each genotype were collected for the ROS or ATP measurements. Experiments were performed with three independent pools of animals.

We have reported that *Wrn^Dhel/Dhel^* mice exhibit increased ROS and decreased ATP levels in different tissues compared to age-matched wild type animals [16, 34]. To determine whether the loss of *wrn-1* also affects ROS levels in *C. elegans*, we measured ROS levels in whole *wrn-1(gk99*) worms with dichlorofluorescein (DCFA) staining as described previously [34]. Although not significant, the *wrn-1(gk99)* mutant worms exhibited an 8% increase in overall ROS levels compared to the wild type strain (Figure 3A). The loss of *mir-124*, in return, led to a significant increase in overall ROS levels (16% increase; *P* = 0.0442). Interestingly, the loss of both *wrn-1* and *mir-124* resulted in a 40% increase in whole body ROS levels compared to wild type worms (*P* = 0.0008) (Figure 3A).

We next measured the impact of the loss of *wrn-1* and/or *mir-124* on ATP levels. The *wrn-1(gk99)* animals exhibited a 35% decrease in overall ATP levels compared to the wild type strain (Figure 3B), while the *mir-124(n4255)* animals exhibited a 52% decrease in the overall ATP levels compared to the wild type strain (*P* = 0.0409). Finally, the double mutant *wrn-1;mir-124* worms showed a 63% decrease in whole body ATP levels compared to wild type animals (*P* = 0.0137). These results indicate that the loss of both wrn-1 and mir-124 functions significantly affect ATP levels in *C. elegans*.

### The loss of *wrn-1* and *mir-124* lead to an increase of the aging marker lipofuscin

**Figure 4:**
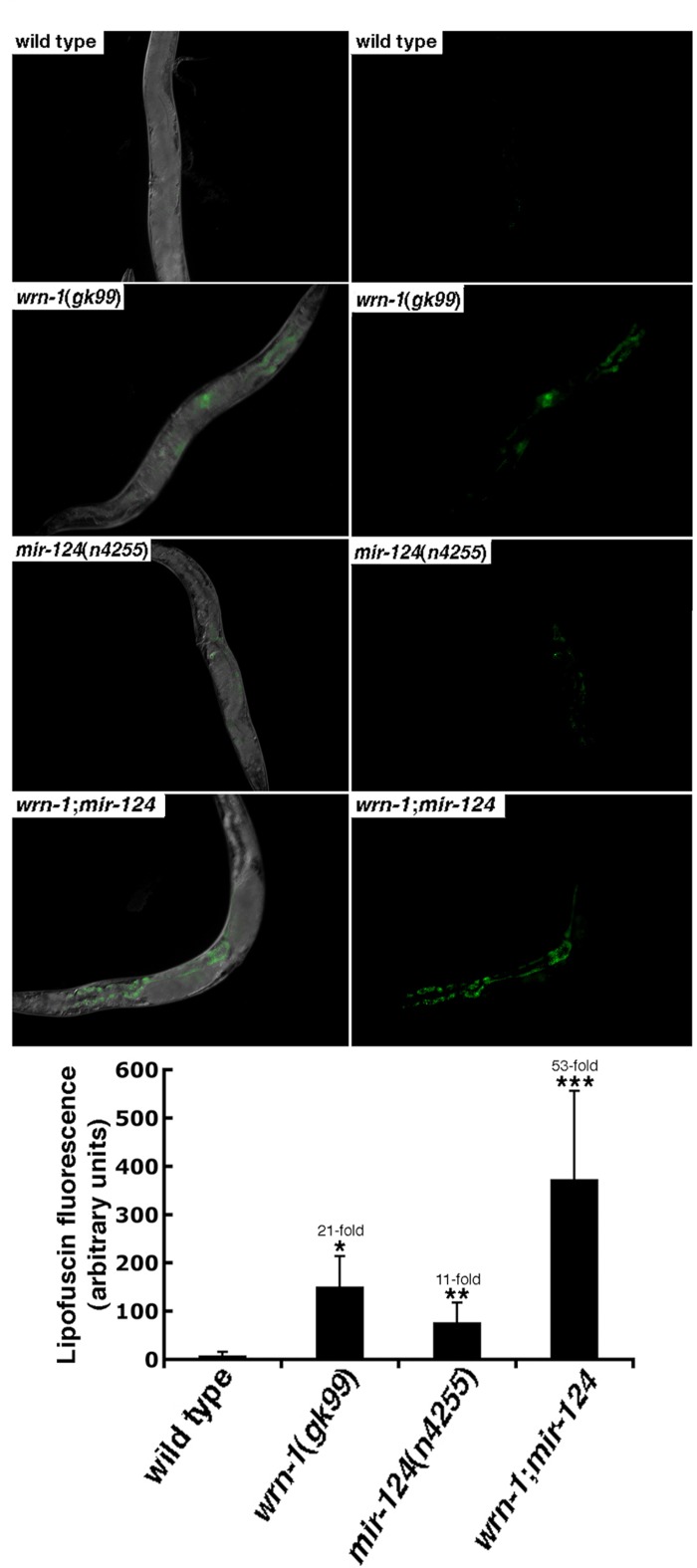
The aging marker lipofuscin is increased in mutant *C. elegans* strains. Representative photographs of wild type (N2), *wrn-1(gk99), mir-124(n4255)*, and *wrn-1;mir-124* double mutant worms at three days into adulthood. Panels on the right represent the lipofuscin autofluorescence alone. All pictures were taken at the same exposure time. Magnification is 10 X. The histogram at the bottom represents the average intensity of lipofuscin autofluorescence in the different *C. elegans* strains. Ten to fifteen three-days old (three days into adulthood) worms of each strain were photographed and the fluorescence intensity was quantified using Adobe Photoshop. The fold increase in fluorescence intensity compared to wild type animals is indicated. (Unpaired Student's t-test; *P = 0.00002 for *wrn-1(gk99)* vs. wild type; **P = 0.00222 for mir-124(n4255) vs. wild type; and ***P = 0.00078 for *wrn-1;mir-124* vs. wild type).

To determine whether the reduced life span observed in*mir-124(n4255)* worms was due to a progeroid phenotype, accumulation of the aging marker lipofuscin was examined. The intensity of the fluorescence observed in the *wrn-1(gk99)* animals was 21-fold stronger than wild type worms at the third day into adulthood (Figure 4). Similarly, the lipofuscin fluorescence observed in *mir-124(n4255)* mutant worms was also stronger than in the wild type animals. Finally, there was a synergic effect on the accumulation of lipofuscin in the double mutant *wrn-1;mir-124* worms (Figure 4). These results indicate that the animals lacking either *wrn-1* or *mir-124* exhibit a progeroid phenotype that is exacerbated by the loss of both genes.

### Vitamin C restores the normal life span of *wrn-1(gk99)* and *mir-124(n4255)* mutant strains

**Figure 5:**
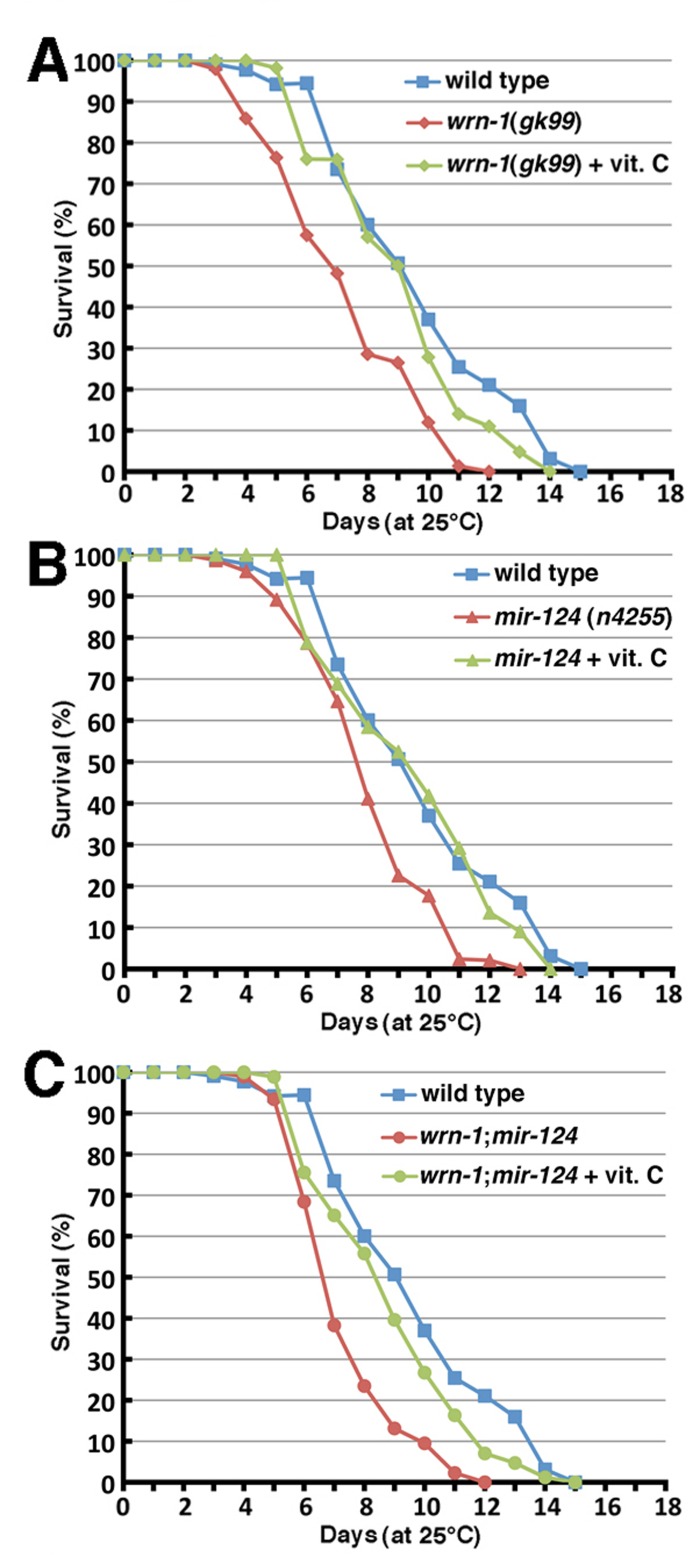
Impact of vitamin C on the life span of wild type and mutant *C. elegans* strains. (**A**) Survival curves of vitamin C treated *wrn-1(gk99)* and of untreated wild-type (N2) and *wrn-1(gk99) C. elegans* strains grown at 25°C (vitamin C treated *wrn-1(gk99)* vs. untreated *wrn-1(gk99)* worms: *P* = 1.4 × 10^−7^; vitamin C treated *wrn-1(gk99)* vs. untreated wild-type worms: *P* = 0.0778). (**B**) Survival curves of vitamin C treated *mir-124(n4255)* and of untreated wild-type (N2) and *mir-124(n4255)C. elegans* strains grown at 25°C (vitamin C treated *mir-124(n4255)* vs. untreated *mir-124(n4255)* worms: *P* = 3.0 × 10^−9^; vitamin C treated *wrn-1(gk99)* vs. untreated wild-type worms: *P* = 0.359). (**C**) Survival curves of vitamin C treated *wrn-1;mir-124* and of untreated wild-type (N2) and *wrn-1;mir-124* double mutant *C. elegans* strains grown at 25°C (vitamin C treated *wrn-1;mir-124* vs. untreated *wrn-1;mir-124* worms: *P* = 3.1 × 10^−6^; vitamin C treated *wrn-1;mir-124* vs. untreated wild-type worms: *P* = 0.0163). All experiments were performed three to four times with 20 to 30 worms per genotype. *P*-values were obtained using the log-rank test method.

Previously, we reported that vitamin C restored the normal life span of *Wrn^Dhel/Dhel^* mice [16]. We thus decided to test the impact of 10 mM ascorbate [35] on the life span of each *C. elegans* mutant strain. Vitamin C significantly increased the median life span of *wrn-1(gk99)* animals when they were grown with a diet containing vitamin C (log-rank test: *P* = 1.4 × 10^−7^; Figure 5A). Furthermore, this lifespan extension effect was comparable to wild type animals grown on normal media. The median life span of *mir-124(n4255)* mutant worms was also significantly increased to a level similar to wild type animals upon vitamin C supplementation (Figure 5B; log-rank test: *P* = 3.0 × 10^−9^). These results indicate that vitamin C significantly increased the life span of animals lacking the *wrn-1* or *mir-124* genes.

Finally, we determined the life span of double mutant *wrn-1;mir-124* worms treated with vitamin C. While vitamin C did extend the lifespan of these double mutant worms from 6.6 days to 8.4 days (Figure 5C, *P* = 3.1 × 10^−6^), it was not as a dramatic effect as observed for the single mutant worms. Furthermore, vitamin C treatment did not increase the life span of the double mutants to that of the untreated wild type worms (Figure 5C; log-rank test: *P* = 0.0163).

### Vitamin C decreases ROS levels in all mutant strains

**Figure 6:**
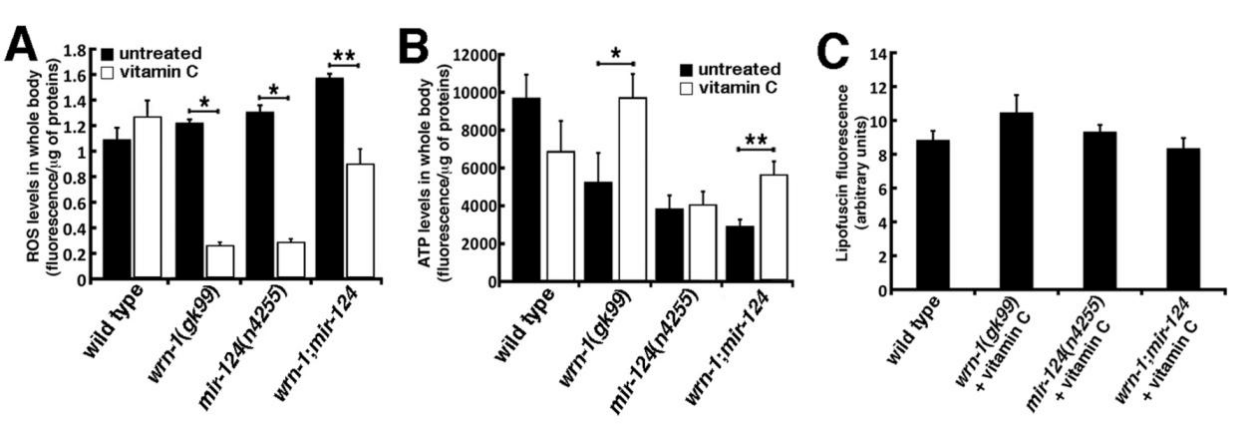
Impact of vitamin C on ROS, ATP, and lipofuscin levels in wild type and mutant *C. elegans* strains. (**A**) ROS levels in whole body of 7-day adult old worms (post-larval L4 stage) detected with dichlorofluorescein. Data are expressed as units of fluorescence per milligrams of proteins. (Unpaired Student's t-test; *P < 0.00005; ** *P* = 0.00009). (**B**) ATP levels in whole body of 7-day old adult worms (post-larval L4 stage). (Unpaired Student's *t*-test; *P = 0.00972; **P = 0.0002). Twenty-five worms of each genotype were collected for the ROS or ATP measurements. Experiments were performed in triplicate. (**C**) Histogram representing the average intensity of autofluorescence lipofuscin in the different *C. elegans* strains treated with vitamin C compared to untreated wild type worms. Ten to fifteen three-days old (three days into adulthood) worms of each strain were photographed and the fluorescence intensity was quantified using Adobe Photoshop.

We next examined the effect of vitamin C on ROS levels in whole worms of each strain. Twenty-five 7-day old adult worms (timed from the post-larval L4 stage) of each genotype were treated with 10 mM vitamin C and then ROS levels were measured. There was no significant difference between untreated and vitamin C-treated wild type worms. In *wrn-1(gk99)* and *mir-124(n4255)* worms, vitamin C treatment significantly lowered ROS levels compared to untreated worms (Figure 6A; *P* < 0.00005). Finally, vitamin C also significantly decreased ROS levels *in wrn-1;mir-124* worms compared to the untreated *wrn-1;mir-124* animals (*P* = 0.00009) (Figures 6A). Overall, these results indicate that vitamin C significantly decreased ROS levels in all mutant strains tested.

### Vitamin C increases ATP levels only in the *wrn-1(gk99)* mutant strain

We also measured ATP levels in vitamin C treated mutant worms. ATP levels were decreased in wild type treated worms compared to untreated wild type worms but this decrease was not statistically different. ATP levels in vitamin C-treated *wrn-1(gk99)* worms were similar to the ATP levels of untreated wild type worms (Figure 6B). In contrast, vitamin C significantly increased ATP levels in *wrn-1(gk99)* worms compared to the untreated *wrn-1(gk99)* animals by 1.9-fold (*P* = 0.00972; Figure 6B).

ATP level was not significantly increased in vitamin C treated *mir-124(n4255)* animals compared to the untreated *mir-124(n4255)* worms (Figure 6B) and was still at a lower level than untreated wild type animals (*P* = 0.0155). Thus, vitamin C did not normalized the amount of ATP in *mir-124(n4255)* worms to the wild type levels. There was a 1.9-fold increase in ATP levels in vitamin C treated *wrn-1;mir-124* double mutant worms compared to untreated *wrn-1;mir-124* animals (*P* = 0.0002; Figure 6B). However, the amount of ATP in vitamin C treated *wrn-1;mir-124* double mutant worms was still lower than untreated wild type animals (*P* = 0.0440). Overall, these results indicate that vitamin C significantly increased ATP levels only in worms bearing the *wrn-1(gk99)* mutation.

### Vitamin C decreases lipofuscin levels in all mutant strains to the level of untreated wild type animals

The intensity of autofluorescence from lipofuscin accumulation was examined in all the mutant strains treated with vitamin C. As indicated in Figure 6C, vitamin C decreased the intensity of autofluorescence in all the mutant strains to untreated wild type levels. These results indicate that vitamin C normalized lipofuscin accumulation in *wrn-1(gk99), mir-124(n4255)*, and *wrn-1;mir-124* worms.

## DISCUSSION

### Important parallels between mouse and *C. elegans* with a mutation in the *WRN* helicase

In this study, we have demonstrated that a *C. elegans* animal carrying a deletion of the wrn-1 helicase have a reduced life span, and importantly this phenotype is similar to mice lacking the DNA helicase activity of the human *WRN* ortholog [16, 30, 34]. Thus, both models can be used to identify and assess the impact of specific genes that, with the *WRN* orthologs, affect health or life span. The short life span of the *C. elegans* allows a rapid evaluation of the impact of a gene on aging, which can then be translated to a more complex organism like the mouse. In this study, we identified *miR-124* as a conserved miRNA in both mouse and worm animal models. miR-124 has a role in premature aging through the loss of a functional WRN ortholog helicase activity, although the mechanism by which the loss of WRN affects miR-124 expression remains somewhat unknown. Nevertheless, we demonstrate that a deletion of the *mir-124* gene ortholog in *C. elegans* results in reduced life span, increased whole body ROS levels, and reduced ATP levels. Because total inactivation of both *wrn-1* and *mir-124* genes had a greater negative impact on ROS and ATP levels than inactivating *wrn-1* alone, these results suggest that the decrease of the miR-124 miRNA can contribute to several key biological processes affected in *Wrn^Dhel/Dhel^* mice [15, 16, 34]. In addition, the deletion of *mir-124* accelerated the accumulation of the aging marker lipofuscin in *C. elegans* and thus highlights the importance of this miRNA in the progeroid phenotype.

The expression of *miR-124* was not only reduced in the livers of young *Wrn^Δ^^hel/^^Δ^^hel^* mice compared to age-matched wild type mice, but it was also reduced in the livers of old wild type mice compared to young wild type mice. These results indicate that the miR-124 expression signature in the liver of young *Wrn^Δ^^hel/^^Δ^^hel^* mice corresponds to the miR-124 signature in old wild type animals. To our knowledge, this is the first study showing a significant altered expression of *miR-124* in the liver of aging mice. Previous studies have not shown an alteration of *miR-124* during normal hepatic aging in mice or rats, or in the long-lived Ames dwarf mice [24, 27, 36]. This difference may be due to the different techniques used for the initial miRNA detection. Previous studies utilized hybridization of labeled molecules on nitrocellulose-based microarray [24, 27, 36] that may be less sensitive than direct quantitative RT-PCR of individual miRNA as was used in this study [37]. Interestingly, the level of miR-124 has also been reported to be down regulated in skeletal muscle of old mice compared to young mice [25]. These results, together with our data, indicate that a decrease of miR-124 can be considered as a common signature in the liver and muscle of aging mice. Our observation of a significant decrease in miR-124 levels in aging *C. elegans* further supports the role of this conserved miRNA in the molecular signature of aging in different animal species.

The miR-124 has been shown to be involved in neurogenesis not only in mouse but also in *C. elegans* [38, 39]. More precisely, the expression of miR-124 in the mouse brain is associated with the differentiation status of neuronal cells [38]. However, miR-124 is expressed in cell types other than neurons [40, 41]. Of relevance to our study, miR-124 is also expressed in the normal human liver [42]. As miR-124 is a regulator of several proteins involved in insulin exocytosis and intracellular signaling in pancreatic beta cell lines [40, 41], it is possible that miR-124 may alter insulin action in vivo directly impacting on organismal homeostasis and aging. Importantly, the insulin/insulin-like growth factor-1 signaling pathway is a strong regulator of longevity in *C. elegans* [23, 43, 44]. Noticeably, insulin-like peptides are primarily released from neurons in C. elegans [23]. Thus, the mutant *C. elegans* strains described in this study gives us relevant models to thoroughly decipher the molecular mechanisms involved in WS and aging in general. As miR-124 will affect protein expression by destabilizing RNA levels of target genes or by inhibiting translation of target mRNAs, the next step is to perform large scale proteomic analyses to identify proteins in our *Mus musculus* and *C. elegans* animal models involved in the insulin signaling pathway, redox balance, energy homeostasis, and healthy aging.

**Vitamin C normalizes the life span of mutant *wrn-1* and *mir-124* strains** We recently found that Vitamin C supplementation rescued the shorter mean life span of *Wrn^Dhel/Dhel^* mice and reversed several age-related abnormalities in adipose, cardiac, and liver tissues [16]. In this study, we show that vitamin C also rescued the shorter life span of both *wrn-1(gk99)* and the *mir-124(n4255)* mutant animals. Furthermore, vitamin C reversed the increased ROS levels, the decreased ATP levels, and the accelerated accumulation of the progeroid marker lipofuscin in both mutant strains. Lipofuscin is believed to be a mix of oxidized and cross-linked macromolecules, including proteins, lipids, and carbohydrates [45]. Such results point to metabolic abnormalities in worms lacking the helicase function of the human WRN ortholog like *Wrn^Δ^^hel/^^Δ^^he^*^l^ mice [16, 30, 34]. Importantly, we found that vitamin C reversed the metabolic abnormalities in both of these models.

To conclude, our data indicate that miR-124 is a conserved miRNA that is involved in the aging phenotype across mouse and worm species. Furthermore, the loss of miR-124 expression is associated with the lack of WRN helicase function in both species. Finally, the progeroid phenotypes associated with either WRN or miR-124 mutations can be reversed by vitamin C treatment. Finally, our results with both mouse [16] and worm models of WS suggest that vitamin C supplementation could have beneficial effects for patients with WS.

## METHODS

### MicroRNA expression profiling

Care of mice was in accordance with the guidelines of the Centre de Recherche des Centres Hospitaliers Universitaires de Québec. The TaqMan® Array Rodent MicroRNA Card Set v3.0 is a two card set containing a total of 384 TaqMan® MicroRNA Assays per card. The set enables accurate quantification of 755 unique microRNAs for mouse. Included on each array is three TaqMan® MicroRNA Assay endogenous controls to aid in data normalization and one TaqMan® MicroRNA Assay not related to rodent as a negative control. Use of the Megaplex™ RT Primers, Rodent Pool Set v3.0 was required to run the array sets. An additional preamplification step was carried out with Megaplex™ PreAmp Primers. Reactions were performed on four animals, two for each genotype, and according to the manufacturer's protocol (Applied Biosystems, Carlsbad, CA). Raw CTs were then successively normalized using the endogenous U6 and quantile normalization. An empirical Bayesian method within the package limma in BioConductor (http://www.bioconductor.org) was used to identify the significantly modulated miRNAs. A miRNA was judged significantly modulated if the Benjamini-Hochberg adjusted P-value was lower than 0.1. All miRNA analyses were performed using R version 2.14.0.

### Validation of miRNA expression

The quantitative measure of selected miRNA expressions was performed with TaqMan MicroRNA assays on extracted total RNA from four different *Wrn^Dhel/Dhel^*mutant and four wild type mice or on extracted total RNA from four young (three months) and four old (21 months) wild type mice following manufacturer's protocol (Life Techonology, USA).

### *Caenorhabditis elegans* strains

All *C. elegans* strains were maintained as described [46]. Both *wrn-1(gk99)* and *mir-124(n4255)* strains obtained from the *C. elegans* Genetics Center (University of Minnesota, St Paul, MN) were out-crossed four times with the wild type N2 strain to remove possible unrelated mutations. The *wrn-1(gk99)* contains a 196 bps deletion that inhibits the expression of the protein [32]. The primers used to genotype this strain are 5’-CTGGCTGTAACT GCACCTGA-3’ and 5’-AAATGGGAGGGAAAGAGC AT-3’. The *mir-124(n4255)* strain contains a 212 bps deletion that spans the entire mir-124 sequence. The *mir-124* sequence is localized in an intron of the *trpa-1* gene. It has been shown that the *n4255* deletion does not abrogate the expression of the *trpa-1* gene in *C. elegans* [39]. The primers used to genotype the *mir-124(n4255)* strain are 5’-TTGCTTCTTCTTCGAGCA CA-3’ and 5’-AAATGGGAGGGAAAGAGCAT-3’.

### Expression of mir-124 in *C. elegans*

Three hundred 7-days old adult worms (post-larval L4 stage) were sorted by size to exclude remaining larvae using a COPAS BIOSORT instrument (Union Biometrica, Inc., Somerville, MA, USA). Sorted worms were spun down in an eppendorf tube and lysed in TRIZOL (Invitrogen, Carlsbad, CA) to extract total RNA. To measure *mir-124* expression, TaqMan Small RNA assays (Applied Biosystems) were performed as described before. Stem-loop qRT-PCR for mature miRNAs was performed on a real-time PCR system (AB 7900; Applied Biosystems). The short nuclear RNA sn2841 were measured and used as an endogenous control.

### Measurement of life span and aging markers

Worms were transferred to fresh plates and were grown at 25°C. Death was scored by absence of any movement after several light pokes with a platinum wire. Lipofuscin was detected as autofluorescence in adult worms and images were captured using a Zeiss motorized Axioplan 2 microscope (with 525 nm filter) equipped with an AxioCam MRm camera and the AxioVision acquisition software (Carl Zeiss Microscopy GmbH, Jena, Germany).

### ATP quantification in *C. elegans*

ATP levels were quantified with the ApoSensor ATP assay kit according to the manufacturer's instruction (BioVision, Mountain View, CA). Luminescence was measured with a Luminoskan Ascent luminometer (Thermo Electron Inc., Milford, MA). Twenty-five 7-days old adult worms (post-larval L4 stage) worms were collected spun in an eppendorf tube and resuspended in 250 mL of assay kit buffer. Worms were crushed in a Dounce homogenizer (25 strokes) and the homogenate was spun 5 min at 13,000 rpm on a bench top centrifuge at room temperature. The ATP level was measured from the homogenate. Protein concentrations were measured using the Bradford assay. Results were expressed as amount of ATP/mg of proteins. All experiments were performed in three independent pools of animals.

### Reactive oxygen species (ROS) quantification in *C. elegans*

ROS quantification was performed on twenty-five 7-days old adult worms (post-larval L4 stage). Worms were collected spun in an eppendorf tube, resuspended in 250 mL of RIPA buffer (50 mM Tris HCl (pH 7.5), 150 mM NaCl, 1% NP-40, 0.1% SDS, 0.5% deoxycholate), crushed in a Dounce homogenizer (25 strokes). The homogenate (150 μl) was incubated with 10 μg/ml of the dye 2’-7’ dichlorofluorescein diacetate (Sigma-Aldrich) for one hour at 37°C. This dye is highly fluorescent upon oxidation. The oxidized dye was measured as described previously [34]. Protein concentrations were measured using the Bradford assay.

### Statistical analysis

Data on graphs are presented as means + SD. The unpaired Student's t-test and the log-rank test were all performed using an alpha level of 0.05 and a two-sided hypothesis. Life span curves were build on differences between strains were considered significant at P-value lower than 0.05 in all statistical analyses. All statistical analyses were performed using R version 2.14.0 (www.r-project.org).

## SUPPLEMENTARY DATA


