# Supplementary material for: Down regulation of miR-124 in both Werner syndrome DNA helicase mutant mice and mutant Caenorhabditis elegans wrn-1 reveals the importance of this microRNA in accelerated aging
Source: Aging (Albany NY). 2012 Sep 29;4(9):636–47. doi: 10.18632/aging.100489 (PMC3492227; doi:10.18632/aging.100489)
Supplement: Supplementary file 1 [file aging-04-636-s001.pdf]

and intracellular signaling in pancreatic beta-cell lines. J Biol Chem. 2007; 282:19575-19588.

41. Lovis P, Gattesco S, Regazzi R. Regulation of the expression of components of the exocytotic machinery of insulin-secreting cells by microRNAs. Biol Chem. 2008; 389:305-312.

42. Hatziapostolou M, Polytarchou C, Aggelidou E, Drakaki A, Poultsides GA, Jaeger SA, et al. An HNF4alpha-miRNA inflammatory feedback circuit regulates hepatocellular oncogenesis. Cell. 2011; 147:1233-1247.

43. Friedman DB, Johnson TE. A mutation in the age-1 gene in Caenorhabditis elegans lengthens life and reduces hermaphrodite fertility. Genetics. 1988; 118:75-86.

44. Kenyon C, Chang J, Gensch E, Rudner A, Tabtiang R. A C. elegans mutant that lives twice as long as wild type. Nature. 1993; 366:461-464.

45. Yin D. Biochemical basis of lipofuscin, ceroid, and age pigment-like fluorophores. Free radical biology & medicine. 1996; 21:871-888.

46. Brenner S. The genetics of Caenorhabditis elegans. Genetics. 1974; 77:71-94.

SUPPLEMENTARY DATA

Please check the full text version for Table S1. Identification of significantly differentially expressed miRNAs from card set A in the liver of Wrn mutant mice compared to wild type mice and Table S2. Identification of significantly differentially expressed miRNAs from card set B in the liver of Wrn mutant mice compared to wild type mice.

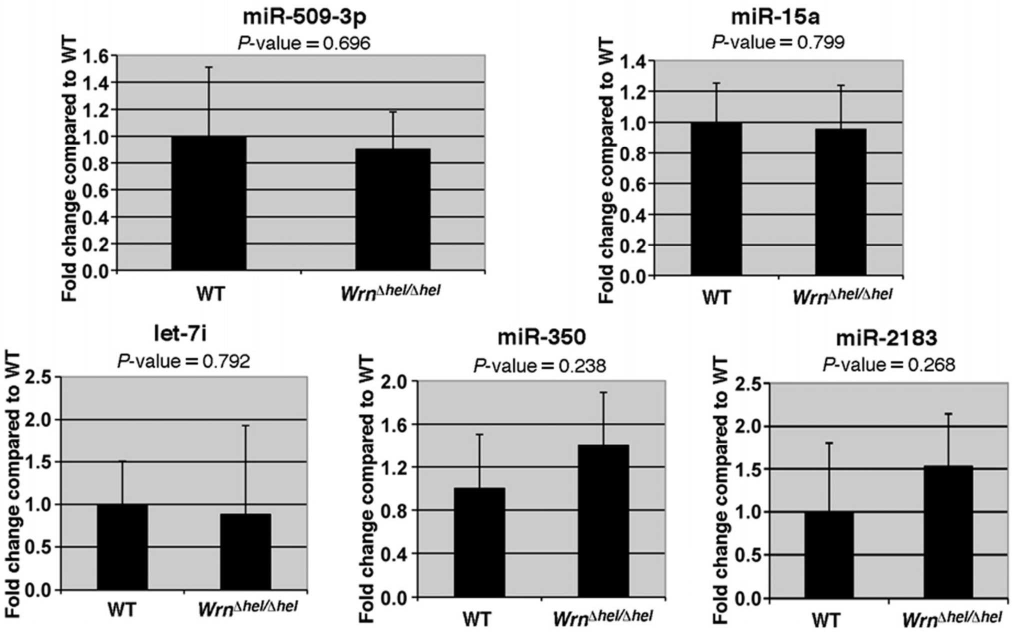

Figure S1. Figure S1. Expression levels of miRNAs from Table 1 in the liver of *Wrn*$^{\Delta hel/\Delta hel}$ mice compared to wild type mice. Total RNA from four mice (at three months of age) of each genotype was used for the quantitative RT-PCR analyses. The levels of the indicated miRNAs in the *Wrn*$^{\Delta hel/\Delta hel}$ mice are relative to the wild type (WT) animals. The P-values (unpaired Student's *t*-test) are also indicated above each graph.
